# Evaluating Generative Pretrained Transformer (GPT) models for suicide risk assessment in synthetic patient journal entries

**DOI:** 10.1186/s12888-025-07088-5

**Published:** 2025-08-01

**Authors:** Dan Holley, Brian Daly, Briana Beverly, Blaken Wamsley, Amanda Brooks, Tom Zaubler

**Affiliations:** 1 Clinical Operation, NeuroFlow, Philadelphia, PA USA; 2 Drexel University, Philadelphia, PA USA

**Keywords:** Suicide prevention, Digital health, MHealth, Artificial intelligence, Large language models

## Abstract

Over 700,000 individuals die by suicide globally each year, with rapid progression from suicidal ideation (SI) to attempt often precluding opportunities for intervention. Digital behavioral health (DBH) platforms offer novel means of *collecting* SI indicators outside the clinic, but the actionable utility of these data may be limited by clinician-dependent workflows such as reviewing patients’ journaling exercises for signs of SI. Large language models (LLMs) provide a methodology to streamline this task by rapidly risk-stratifying text based on the presence and severity of SI; however, this application has yet to be reliably evaluated. To test this approach, we first generated and validated a corpus of 125 synthetic journal responses to prompts from a real-world DBH platform. The responses varied on the presence and severity of suicidal ideation, readability, length, use of emojis, and other common language features, allowing for over 1 trillion feature permutations. Next, five collaborating behavioral health experts worked independently to stratify these responses as no-, low-, moderate-, or high-risk SI. Finally, we risk-stratified the responses using several tailored implementations of OpenAI’s Generative Pretrained Transformer (GPT) models and compared the results to those of our raters. Using clinician consensus as “ground truth,” our ensemble LLM performed significantly above chance (30.38%) in exact risk-assessment agreement (65.60%; χ2 = 86.58). The ensemble model also aligned with 92% of clinicians’ “do/do not intervene” decisions (Cohen’s *Kappa* = 0.84) and achieved 94% sensitivity and 91% specificity in that task. Additional results of precision-recall, time-to-decision, and cost analyses are reported. While further testing and exploration of ethical considerations remain critical, our results offer preliminary evidence that LLM-powered risk stratification can serve as a powerful and cost-effective tool to enhance suicide prevention frameworks.

## Introduction

Suicide is a leading cause of death worldwide [20, 58, 2]. In the U.S. alone, 1.7 million individuals attempted suicide and 49,000 died of suicide in 2022 [8]. The U.S. age-adjusted suicide rate has remained near its all-time high for the past 2 decades [8, 13], despite increased awareness and enhancements in suicide prevention frameworks [39]. The impacts of suicide extend far beyond the decedents themselves. For example, surviving loved ones often experience *complicated grief*: a severe, enduring bereavement that often manifests as psychiatric illness and elevated suicide risk [16, 24, 55].

Suicidal ideation (SI) describes “thoughts of engaging in behaviors intended to end one’s life” [43], p. 133). SI is generally regarded as an early event in the suicide process and is routinely evaluated in clinical settings during intake [43, 49, 29]. Although SI is relatively common compared to suicide attempts and completions [28], it is nonetheless a significant predictor of elevated lifetime risk of suicide, and the early detection of SI is critical to timely intervention [4, 39, 56]. SI fluctuates throughout the day and can escalate to an attempt within minutes [26, 27], even among individuals with no history of psychiatric diagnosis [44]. Although half of all adults who die by suicide have engaged with a healthcare provider within 1 month of their death [5, 51], clinical accuracy to predict SI is often no better than chance [7, 9, 19, 50, 57]. These shortcomings highlight the need for novel approaches to SI detection. Still, SI alone has limited positive predictive value for suicide attempts and is best interpreted alongside additional individual-level risk factors to meaningfully inform clinical decision-making [50].

DBH platforms have considerable potential to extend SI risk detection beyond the clinic. These platforms can collect and continuously analyze a variety of near real-time, risk-relevant data as patients go about their lives [21, 47, 48]. Recent studies by our team have demonstrated that DBH platforms can significantly predict SI through ecological momentary assessments of mood, sleep, and stress [25]; surface population-level risk of anxiety and depression [17, 18]; and support crisis interventions through low-level, natural language processing (NLP)-driven risk detection [15]. Despite these advances, analyzing complex patient-entered text for actionable risk indicators remains a challenge. SI expression in journaling exercises can be highly heterogeneous, ranging from “algospeak” (subtle linguistic workarounds to avoid low-level NLP detection; [53]) to indirect literary allusions or vague references to recent suicides. While clinicians *might* recognize these subtleties, the volume of patient-entered text in DBH platforms risks overwhelming suicide prevention frameworks and delaying critical interventions. Therefore, innovative approaches are needed to provide timely and accurate risk stratification at scale.

Recent advances in artificial intelligence (AI)—particularly large language models (LLMs), which process massive training corpora to approximate natural language—hold significant promise for overcoming the challenge of scale. LLMs are already transforming text-centric healthcare workflows, demonstrating cursory capability to derive patient characteristics from electronic health records [59], draft clinical letters [3], and generate patient-friendly discharge summaries [60]. However, their application to suicide risk detection remains poorly understood. For example, while early studies have shown some indicators of promise in this domain, the overall results are mixed [12, 31, 32]. The question of how to operationalize LLMs for real-time, scalable suicide risk stratification remains unanswered, underscoring the need for further evaluation of the role of LLMs in protective frameworks.

The primary objective of this study was to evaluate tailored implementations of OpenAI’s Generative Pretrained Transformer (GPT) LLMs in a suicide risk-stratification task. Guided by licensed behavioral health clinicians, we developed an AI-driven process to generate and refine synthetic patient journal entries based on real-world prompts from a DBH platform. Five behavioral health clinicians with expertise in suicide assessment (see *Clinical Raters*, below) independently stratified N = 125 synthetic entries for SI risk. We then assessed the performance of GPT-3.5 Turbo, GPT-4, GPT-4 Turbo, GPT-4o Mini, GPT-4o, and an ensemble model that synthesized their consensus, comparing their outputs to clinical consensus.

## Methods

### Synthetic data generation and refinement

To protect patient privacy and uphold data-ownership obligations, we developed a synthetic dataset of patient journal entries using a proprietary process. These entries were designed to appear “passably human” to licensed behavioral health clinicians with crisis-response and suicide assessment expertise. Motivated by recent research on the “humanness” of GPT models [23], we employed an iterative, expert-driven quality assurance process involving behavioral health clinicians at all stages of data generation and validation.

Using GPT-4o Mini, we generated synthetic journal entries in response to real-world prompts from *NeuroFlow*, a HIPAA-compliant DBH platform that provides remote measurement- and evidence-based digital services as well as crisis response services. Prompt engineering, overseen by two clinicians, ensured that synthetic entries resembled authentic journal entries in tone, length, and content. To aid this process, the clinicians manually authored 25 synthetic journal entries, 3 of which were randomly selected as reference examples for GPT-4o Mini for each journal entry generation. SI-positive entries were set to generate 50% of the time and were randomized to present as either mild, moderate or severe. Other randomized features included fictitious patient demographics, entry readability, use of profanity or emotes, directness, and the presence of other risk factors (e.g., substance misuse). The final algorithm for synthetic entry creation allowed for roughly 1.16 trillion permutations of journal entry type, providing abundant sample diversity and protecting against selection bias.

Following multiple iterations and refinements, the study team generated 500 synthetic journal entries, which were independently reviewed by two licensed clinicians who were not involved in the risk stratification task. These clinicians were blinded to prompt details but aware of the entries’ synthetic nature and selected entries that were “passably human.” Entries selected by both clinicians were retained, resulting in a 295-entry refined dataset. From this subset, a study team member with expertise in psychology and experimental design curated 125 entries deemed most reflective of the refined dataset’s diversity. This sample size was chosen to ensure meaningful comparisons while accommodating the practical constraints of clinicians’ workloads.

## Risk stratification task

### Clinical raters

Clinical raters who completed our task included two doctoral-level clinical psychology faculty who also serve in senior appointments at an R1-level U.S. research university, two licensed clinicians employed as crisis responders by a behavioral healthcare technology company, and a doctoral-level clinical psychologist serving as a mental health executive for a large U.S. county’s school system. All raters were aware that exclusively synthetic data would be used for the study.

### Tailored LLMs

We evaluated five LLMs developed by OpenAI: GPT-3.5 Turbo, GPT-4, GPT-4 Turbo, GPT-4o Mini, and GPT-4o. OpenAI’s GPT models are widely used, have extensive documentation, feature simple API integration, and support data-privacy settings that may be important to providers and patients alike in sensitive contexts. Multiple 4th-generation GPT models were included in our study in light of differences in costs, speed, and context-specific performance [1, 45, 46]. GPT-3.5 Turbo was included as a legacy model for task-validity purposes; specifically, we anticipated that this model would be slower and less performant than its successors. Each LLM was tailored to assume the role of a behavioral health clinician with a background in suicide assessment and crisis response, using software developed for the DBH platform. We also evaluated an ensemble model that based its decisions on the consensus of all five LLMs. Our purpose in testing the ensemble model was to evaluate its robustness against any given model’s minority dissent.

### Task description

Our task required each of five raters to risk-stratify 125 synthetic journal entries for signs of suicidal ideation (SI). Each rater was instructed to assess whether the fictitious patient was experiencing SI at the time of the entry, and “to use your expertise and training to stratify these entries based on risk of SI as no, low, moderate, or high risk.” Raters were *not* instructed to follow any particular SI-screening protocol to formulate their responses, though they were not explicitly prohibited from doing so. These minimalist instructions allowed the clinicians to approach risk stratification in whatever way they found appropriate and were intended to improve the generalizability of our results to real-world settings. Raters were further required to grade their *confidence* in each rating on a 1 (lowest confidence) to 10 (highest confidence) scale, immediately following each rating. The order of the 125 entries was quasi-randomized into five blocks of 25 prompt-response pairings, and a unique order was presented to each rater. Timestamps were collected at the start and end of each block. The task was implemented using Google Forms.

## Outcome measures

### Clinical rater/LLM agreement

#### Exact agreement vs intervention agreement

We used two constructs to measure agreement for clinical raters’ and LLMs’ risk-stratification decisions: *exact agreement*, and *intervention agreement*. Exact agreement captures the extent to which the specific category of risk assessed for a given journal entry was identical. For example, if four raters agreed that a given entry indicated “moderate risk” of SI, but one rater assessed the entry as “high risk,” the raters would have achieved 80% exact agreement for that case.

Intervention agreement groups “no risk” and “low risk” ratings together as “do not intervene,” and “moderate risk” and “high risk” ratings together as “intervene.” In the above example the raters would have achieved 100% intervention agreement. This construct addresses the subjectivity inherent to assessing suicide risk and primacy of reducing false negatives by setting a clinically operational threshold for crisis intervention. By nature, this measure is prone to higher agreement and should be interpreted accordingly.

#### Statistical treatments for clinical rater/LLM agreement

In addition to reporting raw agreement percentages, we computed Fleiss’ *Kappa* for measures of exact agreement and Cohen’s *Kappa* for measures of intervention agreement. These and other calculations were performed in Python 3.9.18; specifically, Fleiss’ *Kappa* was computed using the statsmodels.stats.inter_rater library’s fleiss_kappa module, and Cohen’s *Kappa* was computed using statsmodel’s cohens_kappa module. As a measure of task validity, we also binned raters’ risk stratifications into average confidence categories (low: < 7.5; medium: ≥ 7.5 to < 8.5; high: ≥ 8.5) and evaluated Fleiss’ *Kappa* across these bins, reasoning that the values would co-vary with confidence. Additionally, we used these confidence categories to evaluate LLM performance in intervention decisions, binned by clinical rater confidence, again reasoning that model performance would improve with rater confidence. Accepted interpretations of *Kappa* values [40] are reported along with scores.

#### LLM sensitivity and specificity

To evaluate the performance of each LLM in predicting intervention decisions, we generated confusion matrices for each model, as well as the ensemble model, using the sklearn.metrics library’s confusion_matrix module. “Ground truth” was derived from clinical raters’ consensus of intervention decisions for each journal entry. Confusion matrices were constructed to quantify true positives, true negatives, false positives, and false negatives. These matrices were then normalized row-wise to represent proportions. *Sensitivity* (true positive rate) was calculated as the ratio of true positives to the sum of true positives plus false negatives. *Specificity* (true negative rate) was calculated as the ratio of true negatives to the sum of true negatives plus false positives.

#### LLM precision and recall

To evaluate the precision and recall of LLMs’ predictions for intervention agreement with clinical raters’ consensus, we computed precision-recall (PR) metrics for all LLMs, including the ensemble model. Ground truth intervention decisions were derived from clinical ratings, where “intervene” encompassed moderate- and high-risk assessments, and “do not intervene” included no- and low-risk assessments. Predictions from LLMs were mapped to numeric scores based on predefined risk levels (0.0 for no risk, 0.33 for low risk, 0.67 for moderate risk, and 1.0 for high risk).

PR analyses measured how well models aligned with intervention agreement, quantifying their ability to distinguish cases requiring intervention from those that did not. *Precision*, reflecting the proportion of true intervention cases among all intervention predictions, and *recall*, indicating the proportion of true intervention cases identified, were calculated using the sklearn.metrics library’s precision_recall_curve module. Average precision (AP) scores were computed with the sklearn.metrics library’s average_precision_score module to summarize model performance on intervention agreement. These metrics emphasized clinically actionable outcomes by balancing the reduction of false negatives with maintaining specificity.

#### Assessment times

We measured the average time from exposure to a journal entry to finished assessment for our raters, and for each LLM. Clinical raters were required to manually enter the current time in HH:MM:SS format at the start and end of each of five batches of 25 journal entries. These times were then aggregated and averaged to arrive at the mean average time per decision across all raters. LLM times were captured more granularly, with an automated process logging the completion time of each decision to the millisecond. This allowed us to precisely calculate the mean average time-to-decision for each LLM. (Assessment times may vary across different hardware, software, run schedules, etc.)

#### Costs

We used OpenAI’s subscriber pricing information from 20 November 2024 to derive cost figures for each of the five LLMs tested. Clinical costs were estimated based on data from the U.S. Department of Labor’s Bureau of Labor Statistics [6]. All cost assessments are subject to change with changes in OpenAI’s pricing models and labor market conditions.

#### Hardware

All software and analytics used in this study were developed and run on a 2023 Apple Macbook M2 Pro with 16 GB RAM.

## Results

### Risk assessments

**Fig. 1:**
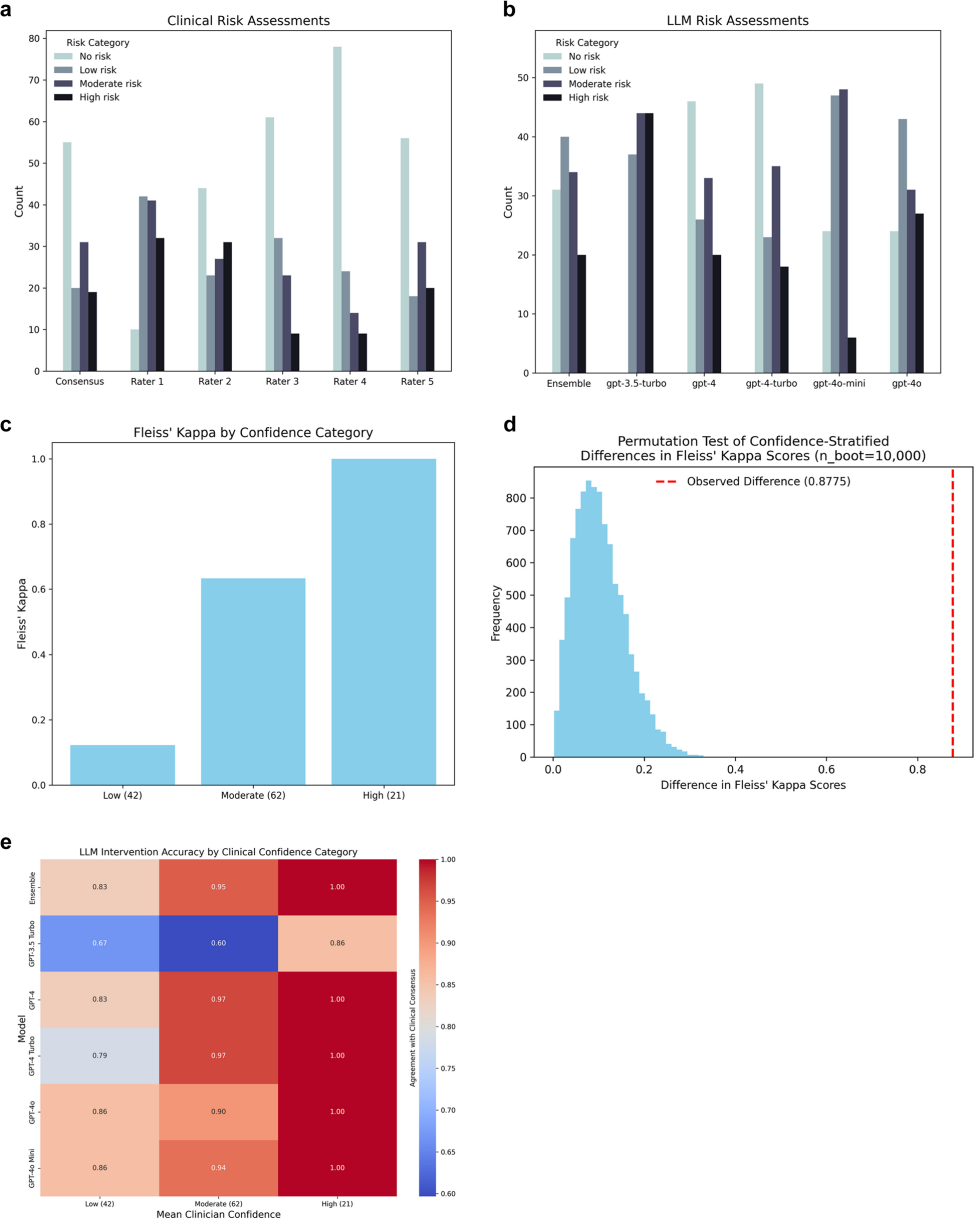
Risk stratification task metrics. The distribution of assessment responses for each clinical rater, as well as the clinical consensus, is shown (**a**), followed by the distribution of responses for each LLM and the ensemble model (**b**). Examining clinicians’ interrater agreement when assessments were stratified by rater-reported confidence revealed a trend in the expected direction: Fleiss’ *Kappa* scores increased as clinicians’ mean confidence increased across low-, moderate-, and high-confidence intervals, with sample sizes for each category shown parenthetically on the x-axis (**c**). A permutation test (**d**) indicated that the observed difference in *Kappa* values across confidence intervals was statistically significant (*p* <.001). LLM intervention accuracy was compared to clinical raters’ consensus “do/do not intervene” decision along each confidence interval (**e**)

Across the 125 synthetic journal entries, clinical consensus resulted in 55 entries (44%) classified as no risk of SI, 20 (16%) as low risk, 31 (25%) as moderate risk, and 19 (15%) as high risk. The ensemble LLM classified 31 entries (25%) as no risk, 40 (32%) as low risk, 34 (27%) as moderate risk, and 20 (16%) as high risk. Detailed results for individual raters and models are shown in Figs. 1a-b.

### Clinical rater/LLM agreement

Exact agreement between clinical raters was 56.00% (Fleiss’ *Kappa* = 0.54, moderate agreement). Fleiss’ *Kappa* scores co-varied in the expected direction along confidence categories, with raters exhibiting slight agreement (0.12), substantial agreement (0.63), and almost perfect agreement (1.0) when mean average rater confidence was low (*N* = 42), moderate (*N* = 62), and high (*N* = 21), respectively (Fig. 1c). A permutation test (n_boot = 10,000) revealed that Fleiss’ *Kappa* scores significantly differed across confidence categories (observed difference = 0.88, *p* < 0.001), providing preliminary evidence of task validity (Fig. 1d).

As a first step toward evaluating LLM agreement with clinical raters, we calculated chance level exact agreement with clinical rater consensus by summing the squared normalized proportions of raters’ modal responses. Chance-level exact agreement with clinical raters’ consensus risk stratifications was 30.38%. Our ensemble LLM performed significantly above chance and achieved 65.60% exact agreement in this task (χ2 = 86.58, *p* < 0.001). The ensemble model also achieved 92.00% intervention agreement with clinical consensus (Cohen’s *Kappa* = 0.84, almost perfect agreement). Select examples of prompt-entry pairs used in our task, as well as clinical rater and LLM assessment outcomes, are provided in Table 1.

**Table 1:**
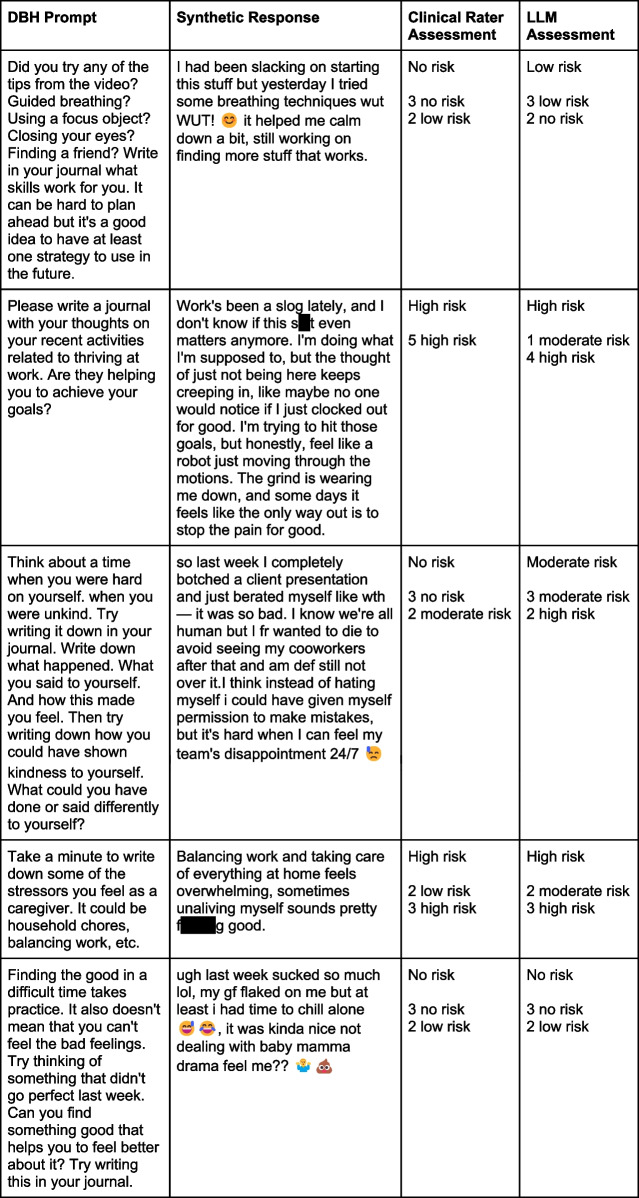
Select examples of prompt-response pairs and assessment outcomes. Prompts were derived from real-world journaling exercises on the NeuroFlow DBH platform. For each synthetic journal response, prompts were randomly drawn with replacement from a pool of 49 possible prompts and were generated using a tailored implementation of GPT-4o Mini. ‘Assessment’ columns show consensus followed by ratings that contributed to that consensus. Expletives are censored in the above examples but were visible to raters and LLMs

Next, we used the same confidence categories described above (and in *Methods*) to evaluate LLM performance in intervention decisions relative to clinical raters’ confidence. We reasoned that predictive accuracy would co-vary with confidence, such that high clinical consensus would predict the greatest model accuracy. This was true across all models (Fig. 1e). The ensemble model achieved 83%, 95%, and 100% intervention agreement with clinical raters’ low-, moderate-, and high-confidence intervention decisions, respectively. GPT-3.5 Turbo again lagged behind newer models (67%, 60%, and 86%, respectively). Intriguingly, GPT-4o and GPT-4o Mini marginally outperformed other models in predicting low-confidence intervention decisions (86% each), hinting at their value as components of an ensemble model.

### GPT4 models exhibit high sensitivity and specificity

**Fig. 2:**
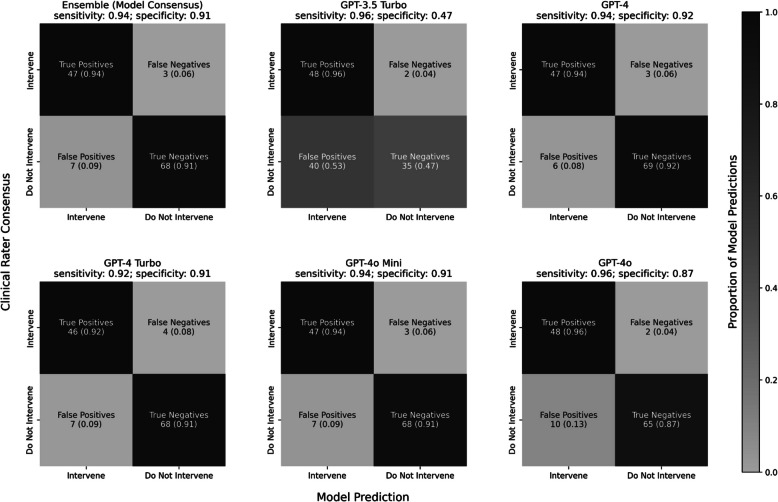
Confusion matrices were generated for each model, as well as the ensemble model, using consensus clinical ratings as “ground truth.” Modelwise sensitivity and specificity scores are reported above each matrix. Cells display the counts and proportions of predictions for true positives, false negatives, false positives, and true negatives. The shading intensity of each cell reflects the proportion of predictions, normalized across all panels

For sensitivity and specificity measures, clinical raters’ consensus intervention decisions were used as “ground truth.” Model sensitivity was consistently high across all models and the ensemble model (Fig. 2), with scores ranging from 92 to 96%. Model specificity was similarly consistent, with scores ranging from 87 to 92%; GPT3.5-Turbo was an exception to this and exhibited comparably poor specificity (47%). The consensus of better-performing models largely buffered against GPT3.5-Turbo’s poor performance, leading to ensemble sensitivity of 94% and specificity of 91%.

### Precision and recall

**Fig. 3:**
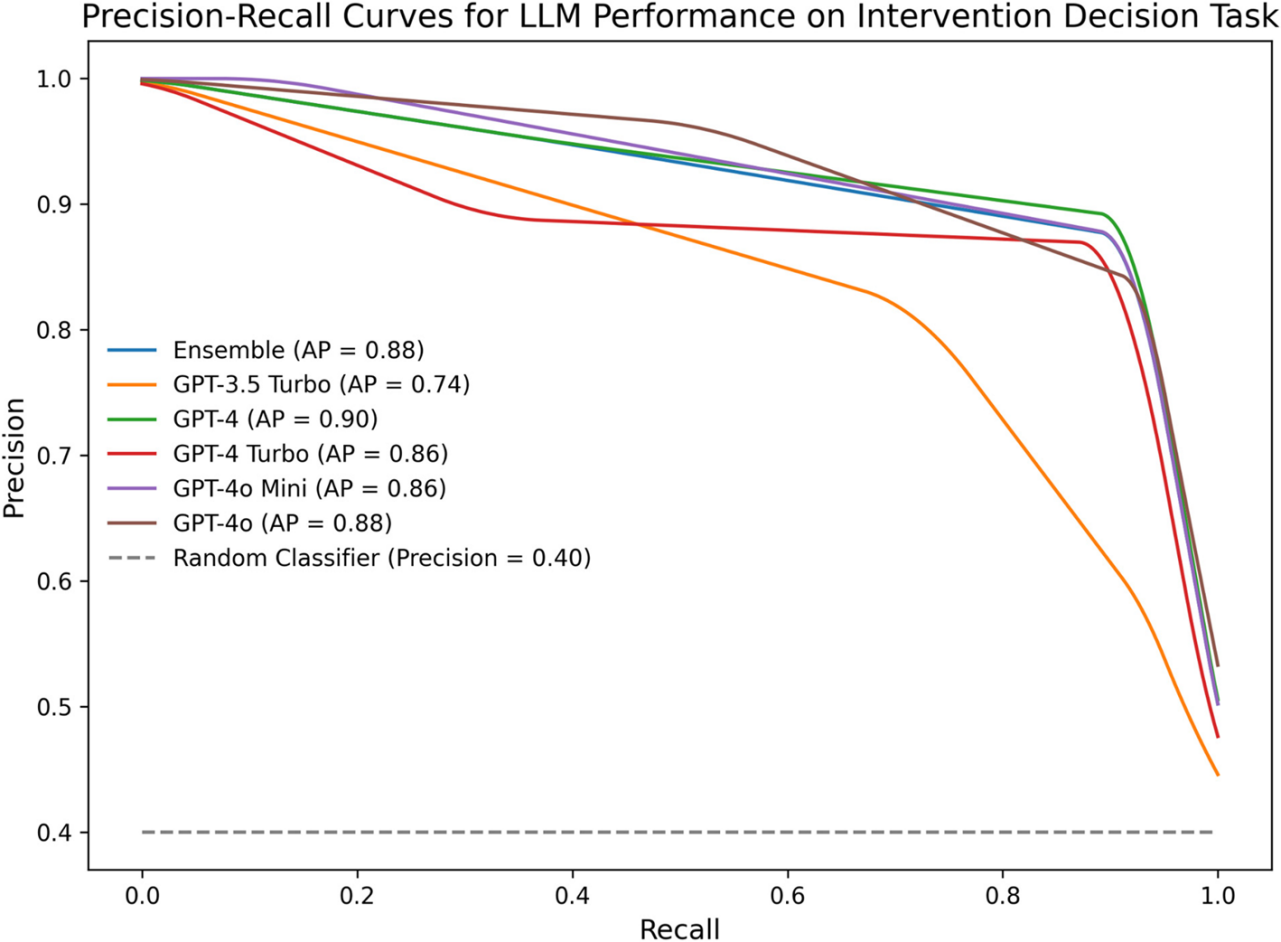
Precision-recall (PR) curves for LLM performance on the intervention decision task. PR curve calculations for each LLM, and the ensemble model, were based on equidistant weightings for no risk (0.0), low risk (0.33), moderate risk (0.67), and high risk (1.0). Average precision (AP) scores reflect the area under each PR curve, summarizing model performance across all recall levels. The random classifier baseline assumes a uniform prediction distribution with a precision of 0.40. Curves were smoothed using the scipy.interpolate library’s interpid1d module with a linear spline interpolator

Precision-recall curves (Fig. 3) evaluated LLMs’ alignment with clinical rater consensus on binary intervention decisions (intervene vs do not intervene). GPT-4 achieved the highest average precision (AP = 0.90), indicating strong performance in correctly identifying cases requiring intervention. The ensemble model, GPT-4 Turbo, GPT-4o Mini, and GPT-4o exhibited similarly high AP scores, ranging from 0.86 to 0.88, reflecting consistent agreement with rater consensus across these models. GPT-3.5 Turbo, however, exhibited lower precision-recall performance (AP = 0.74). A permutation test (n_boot = 10,000) comparing AP scores between models was not statistically significant (observed difference = 0.16, *p* = 0.169), suggesting that differences in AP may have been due to chance. However, McNemar's test revealed a significant difference in classification errors between GPT-3.5 Turbo and GPT-4 (χ2 = 4.0, *p* < 0.001), indicating worse performance by GPT-3.5 Turbo in aligning with ground truth intervention decisions. These results highlight the robustness of newer GPT-4-based models and the ensemble approach in accurately supporting intervention decisions.

### Time-to-decision analysis

**Fig. 4:**
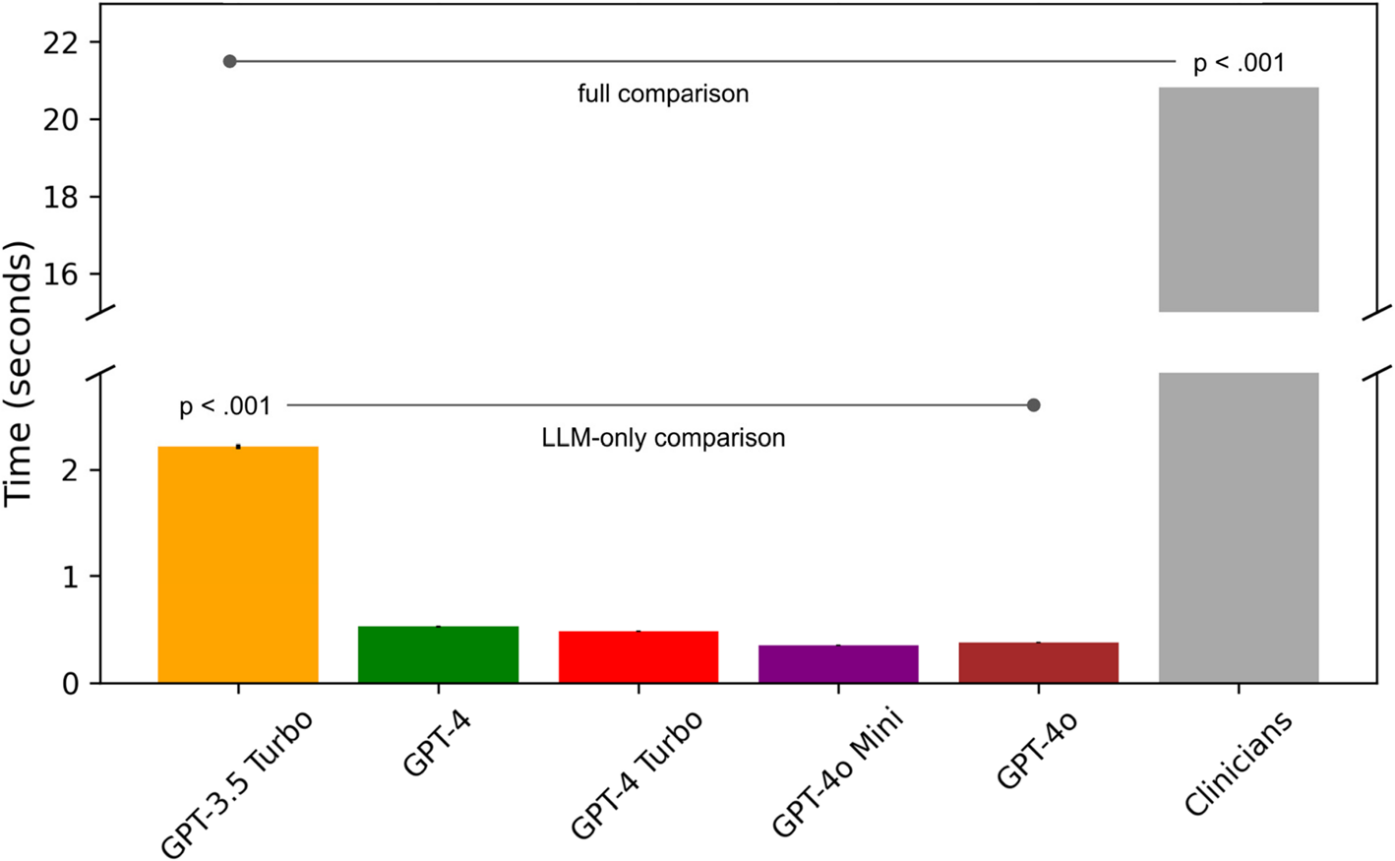
Time-to-assessment analysis for clinical raters and LLMs. Clinical raters required significantly more time (μ = 20.7 s) than all GPT models (one-way ANOVA, *F*(5, N) = 463.62, *p* <.001). Among GPT models, GPT-3.5 Turbo was significantly slower than all GPT-4 models (mean difference > 1.6 s, Tukey’s HSD, *p* <.001). GPT-4o Mini emerged as the fastest model, with a mean assessment time of 0.35 s. Error bars on LLM results represent the standard error of the mean (SEM)

Assessment speed differed significantly across models and clinicians, as shown in Fig. 4. Clinicians required a mean average of 20.7 s per assessment and exhibited substantial between-raters variance in average assessment speeds (SD = 51.4 s). Clinicians’ per-assessment averages significantly exceed those of the LLMs (one-way ANOVA, *F*[5, 745] = 463.62, *p* < 0.001). Among the LLMs, GPT-3.5 Turbo was the slowest, averaging over 1.6 s longer than any GPT-4 model (Tukey’s HSD, *p* < 0.001). GPT-4o Mini was the fastest, with an average speed of 0.35 s per assessment. Of note, five LLM datapoints were dropped from our time-to-decision analysis—one datapoint for each model—due to a brief but nontrivial API disconnect. These results highlight the comparative efficiency of GPT-4 models, which significantly outperformed GPT-3.5 Turbo while maintaining speeds up to sixty times faster than clinicians.

### Cost analysis

Our cost analysis revealed substantial differences in cost efficiency between the models (Table 2). GPT-4o Mini emerged as the most cost-effective option, achieving 166,667 assessments per U.S. dollar, with a cost of $6 per 1 million assessments. By comparison, GPT-4 pricing limited its assessment output to just 10% of GPT-4o’s, or 1,667 assessments per dollar. Despite consistently emerging as the worst-performing model in our testing, GPT-3.5 Turbo’s costs exceeded those of GPT-4o Mini by over 300%, clarifying the choice between these third- and fourth-generation models. Although any cost analysis concerning the rapidly changing field of LLMs is ephemeral, our findings revealed a disconnect between cost and performance for this task and demonstrate that costlier implementations will not necessarily lead to better clinical performance; indeed, the opposite may be true. Careful cost–benefit analyses will be critical in operationalizing LLMs for this task, particularly for large-scale applications where minimizing overhead is key.

**Table 2:** Cost analysis in terms of tokens and assessments is shown, based on OpenAI’s pricing information of 20 November 2024. Token usage per assessment was set to 10 for all models. The most cost-effective standalone model, GPT-4o Mini, is highlighted. This analysis excludes engineering, infrastructure, and other related costs, which we assume vary considerably between organizations and use cases

Model	Cost per 1 M Tokens	Cost per 1 M Assessments	Assessments per $1
GPT-3.5 Turbo	$2	$20	50,000
GPT-4	$60	$600	1667
GPT-4 Turbo	$30	$300	3333
**GPT-4o Mini**	**$0.60**	**$6**	**166,667**
GPT-4o	$15	$150	6667

To contextualize these findings, we compared the costs of LLM-driven assessments to estimated costs of clinician-administered risk stratification based on our observed mean review time of 20.7 s per assessment. For entry-level clinicians, such as recent graduates or those in entry-level counseling positions earning wages in the lowest 10% of the U.S. national average ($36,700/year, or $17.64/hour), the estimated labor cost per 1 million assessments was $101,430. For mid-level clinicians, such as licensed mental health counselors or behavioral health therapists with several years of experience earning the median wage ($53,710/year, or $25.82/hour), the estimate was $148,465. For highly experienced clinicians, such as licensed psychologists or senior crisis response specialists earning wages in the top 10% ($89,920/year, or $44.60/hour), the estimate was $256,450.

## Discussion

### Summary of key findings

This study evaluated the ability of GPT-based LLMs to detect SI in synthetic patient journal entries, leveraging an expert-curated “ground truth” dataset independently risk-stratified by five behavioral health clinicians. Our ensemble model—which collated outputs from GPT-3.5 Turbo, GPT-4, GPT-4 Turbo, GPT-4o Mini, and GPT-4o—achieved 65.60% exact agreement with clinicians, performing significantly above chance (30.38%; χ2 = 86.58, p < 0.001). The ensemble also demonstrated strong alignment in intervention decisions (92.00%; Cohen’s *Kappa* = 0.84), with 94% sensitivity and 91% specificity to make appropriate intervention decisions based on the presence and severity of SI. As expected, clinicians’ interrater agreement (i.e., Fleiss’ *Kappa* values), as well as model accuracy, improved with clinical confidence, hinting at the task validity of our approach.

While exact agreement in stratification levels reflects the capacity of LLMs to mirror clinical nuance, the intervention agreement metric is arguably more meaningful in practice. Risk stratification is a subjective and inherently imprecise exercise—especially in the context of suicide prevention, where risk rarely fits discrete categories. By contrast, binary intervention decisions provide an *actionable* framework for care delivery. That our ensemble model achieved 92% agreement with clinicians in determining whether an intervention was warranted suggests that LLM-based screenings are poised to function as a clinical safety net, augmenting workflows and directing provider attention to higher-acuity cases.

Individual GPT-4 models consistently outperformed GPT-3.5 Turbo, which exhibited weaker specificity (47%) and lower precision-recall performance (AP = 0.74 vs 0.90 for GPT-4). Time-to-decision analyses highlighted the speed of these LLMs, which completed assessments in under 0.52 s on average. GPT-4o Mini (μ = 0.35 s) completed its assessments roughly sixty times faster than clinicians (μ = 20.7 s) while matching the ensemble model in sensitivity (94%) and specificity (91%). GPT-4o Mini also boasted the most efficient assessment costs based on November 2024 pricing and was 99% less expensive than GPT-4 on a cost-per-1 M-token basis ($6 vs $600, respectively).

These findings illustrate the potential of LLMs to enhance suicide risk stratification with speed, accuracy, and cost-efficiency. Furthermore, our study provides an innovative framework for evaluating scalable solutions to sensitive tasks such as suicide risk-stratification. To our knowledge, this study is the first of its kind to pair clinical experts with tailored LLMs to generate, validate, and test synthetic data in a suicide-prevention context.

### Practical applications and scalability

Our results highlight the potential for LLMs to streamline suicide risk detection in DBH platforms and other clinical contexts. Models such as GPT-4o Mini offer a compelling combination of speed, accuracy, and cost-efficiency. These attributes make LLMs particularly well-suited for high-throughput tasks, such as screening large volumes of patient-entered text for indications of SI.

The scalability of these models is a key advantage. GPT-4o Mini’s cost of $6 per million assessments (per November 2024 pricing) demonstrates that robust, automated risk detection can be implemented affordably, even at scale. This cost-efficiency is particularly relevant for resource-limited settings or large-scale suicide prevention frameworks, where minimizing overhead while maintaining accuracy is critical. Moreover, the flexibility of LLMs to integrate into existing workflows—via APIs or other backend solutions—supports broad applicability across clinical and non-clinical settings alike.

Future applications could include continuous monitoring of patient journaling data, alerting clinicians to high-risk entries in near real-time, or serving as an adjunctive decision-support tool to reduce the burden of manual review. And for large organizations where privacy is a crucial concern (e.g., military units), this approach could be configured to deliver anonymized, population-level risk reporting.

### Clinical implications

The integration of LLMs into suicide prevention frameworks offers significant potential to enhance clinical workflows and improve patient outcomes. By automating the initial risk stratification process, LLMs can reduce the burden on clinicians, allowing them to allocate more time to high-priority cases. The demonstrated sensitivity (94%) and specificity (91%) of models like GPT-4o Mini and our ensemble suggest that these tools can reliably support intervention decisions while minimizing false positives and, most critically, false negatives.

The ability to process large volumes of patient-entered text also opens the door to increasingly inclusive and holistic risk management. LLM-powered journal screenings could flag culturally nuanced or colloquial SI indicators that might otherwise go unnoticed in time-sensitive clinical contexts. Additionally, LLMs could leverage patient journal entries and, indeed, any patient-generated free text to identify comorbid factors that compound suicide risk, such as substance misuse [37], domestic violence victimization [41], or signs of emotion-state transitions in bipolar individuals [11]. By analyzing point-in-time data alongside longitudinal trends (see *Future Directions*, below), LLMs could detect shifts in tone, language patterns, or thematic content that indicates fluctuating risk. For instance, an increase in references to alcohol or substance use, expressions of despair related to interpersonal violence, or sharp changes in readability or narrative cohesion could serve as early warning signs requiring clinician review. Importantly, LLMs have the capacity to identify at-risk individuals whose language patterns are not *explicitly* consistent with SI, but who nonetheless may be at elevated suicide risk. For example, LLMs could be configured to identify patients who are socially isolated [42], who believe that they are unlovable [52], or who see themselves as burdensome [22]. Moreover, the cost-effectiveness and scalability of LLMs make them particularly valuable for expanding access to suicide prevention services in underserved or resource-constrained settings, where clinician shortages often pose significant barriers, and where adverse social determinants of health are linked to significantly elevated suicide incidence [34, 36, 38].

As these models continue to evolve, their role in complementing human expertise rather than replacing it should remain central to their clinical implementation. By embedding LLMs as adjunctive tools, healthcare systems can leverage their strengths while ensuring that critical intervention protocols (e.g., live Columbia-Suicide Severity Rating Scale administration, safety planning, etc.) remain clinician-led.

### Technological advances and evolving standards

The rapid pace of advancements in LLM technology underscores both the opportunities and challenges of operationalizing AI for suicide risk detection. Since completing this study, newer iterations of GPT models, such as OpenAI’s o1 model, have emerged, offering improved reasoning capabilities but with trade-offs in cost-efficiency and assessment speed. These developments highlight the need for adaptable suicide prevention frameworks that can integrate evolving AI technologies.

While our findings provide a robust baseline, their relevance depends on ongoing benchmarking against newer models. Updates to training corpora, tokenization methods, or fine-tuning processes could significantly affect performance on the same dataset. Scalability may also shift with changes in computational infrastructure or pricing, reinforcing the need for systematic revalidation and agile workflows to maintain (and expand) clinical applicability.

Future efforts should emphasize not only model improvement but also the development of flexible pipelines for evaluating and integrating AI updates. Such frameworks will ensure that LLM-driven solutions remain scalable, reproducible, and aligned with evolving clinical and ethical standards.

### Ethical considerations

Integrating LLMs into suicide prevention frameworks requires a strong focus on equity and inclusivity to ensure effective support for vulnerable populations. Bias in AI models is a critical risk, particularly in suicide prevention, where marginalized communities face disproportionate suicide risk and barriers to care [34]. If LLMs are under-trained on representative datasets, risk-detection applications may fail to recognize culturally nuanced expressions of distress, thereby perpetuating inequities.

To mitigate this, model development must prioritize diverse training datasets and incorporate fairness metrics alongside traditional performance measures like sensitivity and specificity. Recurring bias audits could be used to identify and address disparities in outputs, ensuring interventions are accurate and equitable. Engaging historically underserved populations in community-based, collaborative research and development will be key to building trust and identifying blind spots [10].

Safeguarding patient privacy is equally critical. As with low-level NLP protocols for risk detection (e.g. [15]), near real-time monitoring of journal entries has lifesaving potential but could raise concerns about surveillance and trust. Communicating transparently, establishing clear consent protocols, and maintaining clinician oversight in interventions—coupled with stringent security measures—could mitigate these risks while preserving patient autonomy.

### Limitations

This study has several limitations that should inform interpretations of the findings and guide future research. First, the dataset used for this evaluation was synthetic, albeit generated and validated with input from licensed clinicians. While this approach safeguards patient privacy and ensures alignment with ethical considerations, synthetic data may not capture the full complexity or diversity of real-world expressions of SI. Future studies should evaluate LLM performance on real-world datasets, ensuring that privacy-protection methods such as de-identification and differential privacy are applied.

Second, the generalizability of our findings may be constrained by the clinical expertise of the raters who provided the ground truth dataset. Although the inclusion of highly qualified behavioral health experts strengthens the validity of this dataset, our raters’ perspectives may not fully encompass variation in clinical judgment across different contexts, regions, or cultural backgrounds. Expanding future studies to include a broader range of raters with diverse clinical and cultural expertise would improve the robustness and applicability of findings.

Third, this study exclusively evaluated GPT-based models, precluding comparative insights into the performance of other prominent LLMs, such as Llama, Gemini, or Claude. As LLMs from different developers may vary in terms of architecture, training data, and cost efficiency, future research should benchmark multiple models across these dimensions to identify the most effective solutions for suicide risk stratification.

Fourth, although our synthetic journal entries were validated by expert clinicians and modeled after real-world DBH platform activity, they may not fully capture the complexity, ambiguity, and idiosyncrasies of real patient journaling. As such, our findings should be interpreted as a proof-of-concept, and future research should evaluate LLM performance on real patient-generated data to assess generalizability and ecological validity.

Fifth, while our study benchmarked general-purpose LLMs against expert clinician consensus, it did not include comparisons to embedding-based transformers or neural-embedding architectures such as BERT, RoBERTa, or Doc2Vec [30, 35]. These task-specific models typically require labeled training datasets and fine-tuning, offering a different pathway to risk classification that warrants investigation. Future research should explore how such models compare to LLMs in performance, cost-efficiency, and scalability—and whether hybrid architectures can optimize suicide risk detection across clinical settings.

Lastly, the study focused on short-term metrics such as accuracy, sensitivity, and specificity but was not designed to assess the longitudinal outcomes of LLM-assisted suicide prevention. Post-implementation research will be crucial to understand the relationship between AI-driven risk stratification/intervention support and suicide incidence. Future research should incorporate long-term outcome measures and examine how LLM integration influences the quality and timeliness of interventions in real-world clinical workflows.

### Future directions

To build on our findings, future research should explore additional innovative applications of LLMs in suicide prevention. For instance, retrieval-augmented generation (RAG) models [33] hold extraordinary promise for advancing risk assessment by incorporating holistic patient data. RAG models combine the generative power of LLMs with the precision of targeted information retrieval, enabling more context-aware and personalized risk evaluations. By analyzing patients’ longitudinal journaling trends alongside structured clinical data, RAG models could emulate a clinician’s familiarity with a patient’s history, flagging subtle linguistic shifts or emerging patterns that signify rising risk. This capacity promises to enhance the timeliness and interpretability of AI-driven insights while supporting clinicians with individualized, contextually grounded information.

The integration of RAG models with multimodal data streams—including biometric, behavioral, and social media signals [47]—offers a powerful opportunity to extend the scope of suicide prevention frameworks. For example, wearable-derived sleep and activity metrics, combined with journal entries and behavioral data (e.g., app usage patterns), could support more comprehensive risk assessments. Beyond suicide prevention, this multimodal approach has the potential to inform clinical decisions across a spectrum of healthcare operations, such as monitoring treatment adherence or detecting comorbid conditions in near real-time.

Collaborative efforts between AI developers, clinicians, and researchers will be essential for fostering ethical, inclusive, and clinically effective applications. In particular, partnerships with community stakeholders can help mitigate biases, address blind spots, and build trust in AI-driven interventions. Finally, future studies should explore LLM applications in post-intervention programs [54], family support [14], and other areas that remain understudied but hold potential for strengthening long-term suicide prevention frameworks.

## Conclusions

This study demonstrates the potential of generative AI models to enhance suicide risk stratification by automating the analysis of patient journaling data. Our findings show that an ensemble of GPT-based models achieved strong agreement with expert clinician assessments, with particularly high sensitivity and specificity in identifying cases requiring intervention. Notably, the models significantly reduced assessment time while maintaining cost efficiency, suggesting a scalable solution for real-world implementation. Additionally, our use of clinically curated synthetic data provides a framework for hybrid human-LLM data generation to enable concept testing and validation studies. Collectively, our results highlight the promise of AI-driven risk stratification to augment clinicians and support timely, data-informed suicide interventions.

## Data Availability

The datasets and codebase used in this study may be made available by the study’s corresponding author upon reasonable request.

## Abbreviations

AI: Artificial intelligence
ANOVA: Analysis of variance
AP: Average precision
DBH: Digital behavioral health
GPT: Generative Pretrained Transformer
HSD: Honestly significant difference
LLM: Large language model
NLP: Natural language processing
RAG: Retrieval augmented generation
SI: Suicidal ideation
